# *N*′-[1-(2,4-Dioxo-3,4-dihydro-2*H*-1-benzopyran-3-yl­idene)eth­yl]thiophene-2-carbo­hydrazide

**DOI:** 10.1107/S1600536811010907

**Published:** 2011-03-31

**Authors:** Madeleine Helliwell, Despina A. Nasiopoulou, Philip A. Harris, Antigoni Kotali, John A. Joule

**Affiliations:** a The School of Chemistry, The University of Manchester, Manchester M13 9PL, England; b Laboratory of Organic Chemistry, Department of Chemical Engineering, University of Thessaloniki, Thessaloniki 54124, Greece; c GlaxoSmithKline, 1250 South Collegeville Road, P.O.Box 5089, Collegeville, PA 19426-0989, USA

## Abstract

The title compound, C_16_H_12_N_2_O_4_S, was obtained by the condensation of 3-acetyl-4-hy­droxy­coumarin with thien-2-ylcarbonyl hydrazide. The pyran ring adopts a 2,4-dione tautomeric form. The benzopyran ring system is almost coplanar with the thio­phene ring [dihedral angle 0.9 (2)°]. The exocyclic C=C double bond has an *E* geometry. The mol­ecular conformation is stabilized by an intra­molecular N—H⋯O hydrogen bond. In the crystal, inter­molecular N—H⋯O hydrogen bonds link the mol­ecules into chains along the *a* axis.

## Related literature

For the synthesis, characterization and reactions of *N*-acyl hydrazones, see: Kotali (2009 ▶); Kotali *et al.*, (2010 ▶).
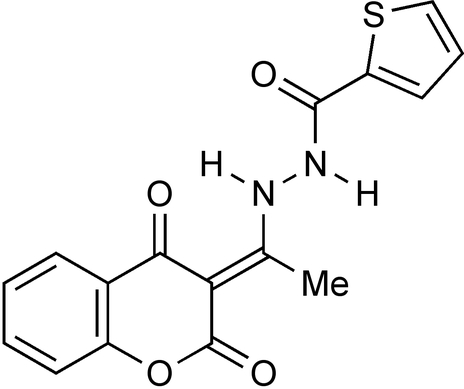


## Experimental

### 

#### Crystal data

C_16_H_12_N_2_O_4_S
*M* *_r_* = 328.34
Triclinic, 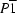

*a* = 4.8631 (11) Å
*b* = 11.833 (3) Å
*c* = 13.296 (3) Å
α = 107.106 (5)°
β = 100.376 (4)°
γ = 97.553 (4)°
*V* = 705.3 (3) Å^3^
*Z* = 2
Mo *K*α radiation
μ = 0.25 mm^−1^
*T* = 100 K
0.55 × 0.15 × 0.08 mm

#### Data collection

Bruker SMART APEX CCD diffractometer
3526 measured reflections
2441 independent reflections
1403 reflections with *I* > 2σ(*I*)
*R*_int_ = 0.072

#### Refinement

*R*[*F* ^2^ > 2σ(*F* ^2^)] = 0.058
*wR*(*F* ^2^) = 0.116
*S* = 0.87
2441 reflections
217 parameters
6 restraints
H atoms treated by a mixture of independent and constrained refinement
Δρ_max_ = 0.33 e Å^−3^
Δρ_min_ = −0.36 e Å^−3^

### 

Data collection: *SMART* (Bruker, 2001 ▶); cell refinement: *SAINT* (Bruker, 2002 ▶); data reduction: *SAINT*; program(s) used to solve structure: *SIR2004* (Burla *et al.*, 2005 ▶); program(s) used to refine structure: *SHELXL97* (Sheldrick, 2008 ▶); molecular graphics: *SHELXTL* (Sheldrick, 2008 ▶) and *PLATON* (Spek, 2009 ▶); software used to prepare material for publication: *SHELXTL* and *PLATON*.

## Supplementary Material

Crystal structure: contains datablocks global, I. DOI: 10.1107/S1600536811010907/rz2570sup1.cif

Structure factors: contains datablocks I. DOI: 10.1107/S1600536811010907/rz2570Isup2.hkl

Additional supplementary materials:  crystallographic information; 3D view; checkCIF report

## Figures and Tables

**Table 1:** Hydrogen-bond geometry (Å, °)

*D*—H⋯*A*	*D*—H	H⋯*A*	*D*⋯*A*	*D*—H⋯*A*
N2—H2*N*⋯O4	1.00 (4)	1.64 (5)	2.481 (4)	140 (4)
N1—H1*N*⋯O1^i^	0.92 (4)	1.93 (4)	2.841 (4)	177 (4)

Symmetry code: (i) 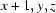.

**Fig. 1.**
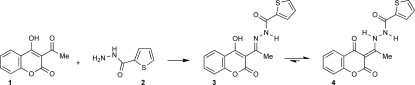
Reaction scheme.

**Fig. 2.**
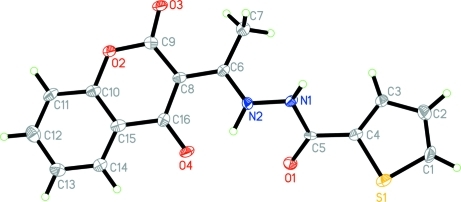
The molecular structure of the title compound with displacement ellipsoids drawn at the 50% probability level.

**Fig. 3.**
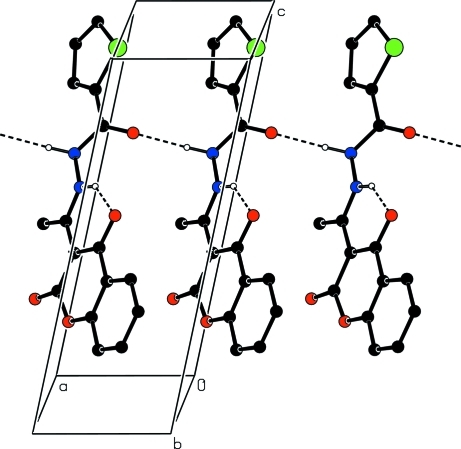
Partial crystal packing of the title compound showing the intra- and intermolecular hydrogen bonds, the latter linking the molecules into one-dimensional chains along the a. H atoms not involved in hydrogen bonding are omitted.

## supplementary crystallographic information

### Comment

In the context of our ongoing studies on the synthesis, characterization and reactions of *N*-acyl hydrazones (Kotali, 2009, Kotali *et al.*, 2010), we reacted 3-acetyl-4-hydroxycoumarin (1) with thien-2-ylcarboxylic acid hydrazide (2) anticipating the formation of the hydrazone (3) (Fig. 1). Spectroscopic measurements strongly suggested that the product adopts the tautomeric form (4). The X-ray determination here described confirmed this hypothesis (Figure 2).

The amide nitrogen, surprisingly, is substantially pyramidal with the sum of the angles of the three substituents amounting to 351.1°. The sum of the angles at the other nitrogen atom, which can be viewed as an enamine nitrogen, is 360.0°. This result illustrates the extensive conjugation between this nitrogen and the two carbonyl groups in the pyran ring *via* the exocyclic double bond. The benzopyran group is essentially coplanar with the thiophene ring, with a dihedral angle of 0.9 (2)°. The exocyclic C═C double bond has *E* geometry. An intramolecular H bond links N2 and O4 (Table 1), and intermolecular H bonds between N1 and O1 link the molecules into one-dimensional chains along the *a* axis (Figure 3).

### Experimental

Thien-2-ylcarboxylic acid hydrazide (1 mmol) was added to a solution of 3-acetyl-4-hydroxycoumarin (1 mmol) in propan-1-ol (20 ml). The mixture was heated at reflux for 24 h and then cooled to room temperature. The resulting precipitate was collected by filtration and dried to give *N*'-[1-(2,4-dioxo-2*H*-1-benzopyran-3(4*H*)- ylidene)ethyl]-thien-2-ylcarboxylic acid hydrazide as a solid (yield 94%). The compound was recrystallizated from propan-1-ol.

### Refinement

H atoms bonded to C were included in calculated positions using a riding model, with aromatic and methyl C—H distances of 0.95 and 0.98 Å, respectively, and U_eq_ values 1.2 and 1.5 times those of the parent atoms; the torsion angles of the methyl H atoms were optimized to give the best fit to the electron density. H atoms bonded to N were found in a difference Fourier map and refined isotropically. The N—H distances are 0.92 (4) and 1.00 (4) Å. Atom C6 was refined subject to an ISOR constraint.

### Crystal data

**Table:**

C_16_H_12_N_2_O_4_S	*Z* = 2
*M**_r_* = 328.34	*F*(000) = 340
Triclinic, *P*1	*D*_x_ = 1.546 Mg m^−^^3^
Hall symbol: -P 1	Melting point = 501–501.5 K
*a* = 4.8631 (11) Å	Mo *K*α radiation, λ = 0.71073 Å
*b* = 11.833 (3) Å	Cell parameters from 557 reflections
*c* = 13.296 (3) Å	θ = 3.3–24.1°
α = 107.106 (5)°	µ = 0.25 mm^−^^1^
β = 100.376 (4)°	*T* = 100 K
γ = 97.553 (4)°	Plate, colourless
*V* = 705.3 (3) Å^3^	0.55 × 0.15 × 0.08 mm

### Data collection

**Table:**

Bruker SMART APEX CCD diffractometer	1403 reflections with *I* > 2σ(*I*)
Radiation source: fine-focus sealed tube	*R*_int_ = 0.072
graphite	θ_max_ = 25.0°, θ_min_ = 1.8°
phi and ω scans	*h* = −5→5
3526 measured reflections	*k* = −9→14
2441 independent reflections	*l* = −14→15

### Refinement

**Table:**

Refinement on *F*^2^	Primary atom site location: structure-invariant direct methods
Least-squares matrix: full	Secondary atom site location: difference Fourier map
*R*[*F*^2^ > 2σ(*F*^2^)] = 0.058	Hydrogen site location: inferred from neighbouring sites
*wR*(*F*^2^) = 0.116	H atoms treated by a mixture of independent and constrained refinement
*S* = 0.87	*w* = 1/[σ^2^(*F*_o_^2^) + (0.0273*P*)^2^] where *P* = (*F*_o_^2^ + 2*F*_c_^2^)/3
2441 reflections	(Δ/σ)_max_ < 0.001
217 parameters	Δρ_max_ = 0.33 e Å^−^^3^
6 restraints	Δρ_min_ = −0.36 e Å^−^^3^

### Special details

**Table:**

Geometry. All e.s.d.'s (except the e.s.d. in the dihedral angle between two l.s. planes) are estimated using the full covariance matrix. The cell e.s.d.'s are taken into account individually in the estimation of e.s.d.'s in distances, angles and torsion angles; correlations between e.s.d.'s in cell parameters are only used when they are defined by crystal symmetry. An approximate (isotropic) treatment of cell e.s.d.'s is used for estimating e.s.d.'s involving l.s. planes.
Refinement. Refinement of *F*^2^ against ALL reflections. The weighted *R*-factor *wR* and goodness of fit *S* are based on *F*^2^, conventional *R*-factors *R* are based on *F*, with *F* set to zero for negative *F*^2^. The threshold expression of *F*^2^ > σ(*F*^2^) is used only for calculating *R*-factors(gt) *etc*. and is not relevant to the choice of reflections for refinement. *R*-factors based on *F*^2^ are statistically about twice as large as those based on *F*, and *R*- factors based on ALL data will be even larger.

### Fractional atomic coordinates and isotropic or equivalent isotropic displacement parameters (Å^2^)

**Table:**

	*x*	*y*	*z*	*U*_iso_*/*U*_eq_	
S1	0.0444 (2)	0.21501 (11)	0.90847 (9)	0.0263 (3)	
O1	−0.1997 (5)	0.2309 (2)	0.68338 (19)	0.0196 (7)	
O2	−0.0262 (5)	0.2667 (2)	0.1854 (2)	0.0191 (7)	
O3	0.2600 (5)	0.1509 (2)	0.2281 (2)	0.0199 (7)	
O4	−0.2081 (5)	0.3697 (2)	0.4843 (2)	0.0189 (7)	
N1	0.1959 (7)	0.1917 (3)	0.6226 (3)	0.0173 (8)	
H1N	0.391 (9)	0.201 (4)	0.640 (3)	0.047 (15)*	
N2	0.0996 (7)	0.2368 (3)	0.5397 (3)	0.0150 (8)	
H2N	−0.014 (9)	0.302 (4)	0.552 (3)	0.048 (15)*	
C1	0.2359 (8)	0.1294 (4)	0.9644 (3)	0.0252 (11)	
H1	0.2486	0.1294	1.0365	0.030*	
C2	0.3682 (8)	0.0614 (4)	0.8950 (3)	0.0262 (11)	
H2	0.4810	0.0070	0.9124	0.031*	
C3	0.3201 (8)	0.0801 (4)	0.7933 (3)	0.0208 (10)	
H3	0.3987	0.0407	0.7351	0.025*	
C4	0.1463 (8)	0.1620 (4)	0.7887 (3)	0.0147 (10)	
C5	0.0318 (8)	0.1993 (3)	0.6974 (3)	0.0125 (9)	
C6	0.1535 (8)	0.1983 (4)	0.4433 (3)	0.0133 (9)	
C7	0.3201 (8)	0.1004 (4)	0.4210 (3)	0.0193 (10)	
H7A	0.5221	0.1358	0.4335	0.029*	
H7B	0.2502	0.0480	0.3456	0.029*	
H7C	0.2976	0.0528	0.4692	0.029*	
C8	0.0388 (8)	0.2517 (4)	0.3664 (3)	0.0141 (9)	
C9	0.1028 (8)	0.2193 (4)	0.2600 (3)	0.0155 (10)	
C10	−0.1924 (8)	0.3525 (4)	0.2107 (3)	0.0176 (10)	
C11	−0.2969 (8)	0.3977 (4)	0.1301 (3)	0.0221 (11)	
H11	−0.2520	0.3710	0.0614	0.027*	
C12	−0.4677 (8)	0.4821 (4)	0.1510 (3)	0.0249 (11)	
H12	−0.5401	0.5138	0.0960	0.030*	
C13	−0.5364 (8)	0.5220 (4)	0.2519 (3)	0.0211 (10)	
H13	−0.6553	0.5797	0.2656	0.025*	
C14	−0.4275 (8)	0.4756 (4)	0.3307 (3)	0.0172 (10)	
H14	−0.4735	0.5015	0.3993	0.021*	
C15	−0.2522 (8)	0.3918 (4)	0.3123 (3)	0.0162 (10)	
C16	−0.1403 (8)	0.3385 (4)	0.3938 (3)	0.0153 (10)	

### Atomic displacement parameters (Å^2^)

**Table:**

	*U*^11^	*U*^22^	*U*^33^	*U*^12^	*U*^13^	*U*^23^
S1	0.0278 (7)	0.0360 (8)	0.0186 (6)	0.0138 (6)	0.0112 (5)	0.0073 (6)
O1	0.0102 (15)	0.0294 (18)	0.0222 (16)	0.0067 (14)	0.0062 (12)	0.0104 (14)
O2	0.0190 (16)	0.0247 (18)	0.0170 (15)	0.0095 (14)	0.0074 (13)	0.0077 (14)
O3	0.0189 (16)	0.0226 (18)	0.0207 (16)	0.0073 (14)	0.0109 (13)	0.0057 (14)
O4	0.0166 (16)	0.0256 (18)	0.0149 (15)	0.0064 (14)	0.0076 (13)	0.0041 (14)
N1	0.010 (2)	0.029 (2)	0.0117 (18)	0.0057 (17)	0.0024 (15)	0.0042 (17)
N2	0.0129 (19)	0.019 (2)	0.0143 (19)	0.0045 (17)	0.0017 (15)	0.0067 (17)
C1	0.021 (2)	0.040 (3)	0.014 (2)	0.005 (2)	0.0002 (19)	0.009 (2)
C2	0.020 (2)	0.036 (3)	0.026 (3)	0.010 (2)	0.004 (2)	0.013 (2)
C3	0.019 (2)	0.031 (3)	0.017 (2)	0.010 (2)	0.0077 (19)	0.010 (2)
C4	0.009 (2)	0.016 (2)	0.013 (2)	−0.0028 (19)	0.0017 (17)	−0.0012 (19)
C5	0.011 (2)	0.011 (2)	0.015 (2)	0.0039 (18)	0.0056 (17)	0.0016 (18)
C6	0.0084 (16)	0.0156 (17)	0.0147 (16)	−0.0025 (13)	0.0041 (13)	0.0043 (14)
C7	0.017 (2)	0.019 (3)	0.021 (2)	0.001 (2)	0.0044 (19)	0.006 (2)
C8	0.010 (2)	0.014 (2)	0.015 (2)	−0.0001 (18)	0.0035 (17)	0.0013 (19)
C9	0.011 (2)	0.014 (2)	0.018 (2)	−0.0019 (19)	0.0017 (18)	0.003 (2)
C10	0.009 (2)	0.023 (3)	0.017 (2)	0.0013 (19)	0.0024 (18)	0.002 (2)
C11	0.026 (3)	0.027 (3)	0.013 (2)	0.005 (2)	0.0081 (19)	0.003 (2)
C12	0.021 (3)	0.026 (3)	0.024 (3)	0.004 (2)	−0.002 (2)	0.008 (2)
C13	0.017 (2)	0.021 (3)	0.022 (2)	0.005 (2)	0.0017 (19)	0.003 (2)
C14	0.013 (2)	0.019 (3)	0.016 (2)	−0.0003 (19)	0.0025 (18)	0.003 (2)
C15	0.010 (2)	0.016 (2)	0.018 (2)	−0.0030 (19)	0.0030 (18)	0.0004 (19)
C16	0.011 (2)	0.017 (3)	0.016 (2)	−0.0026 (19)	0.0025 (18)	0.006 (2)

### Geometric parameters (Å, °)

**Table:**

S1—C1	1.699 (4)	C6—C8	1.423 (5)
S1—C4	1.718 (4)	C6—C7	1.489 (5)
O1—C5	1.232 (4)	C7—H7A	0.9800
O2—C9	1.374 (4)	C7—H7B	0.9800
O2—C10	1.382 (5)	C7—H7C	0.9800
O3—C9	1.223 (4)	C8—C16	1.441 (5)
O4—C16	1.268 (4)	C8—C9	1.454 (5)
N1—C5	1.373 (5)	C10—C11	1.380 (5)
N1—N2	1.395 (4)	C10—C15	1.390 (5)
N1—H1N	0.92 (4)	C11—C12	1.380 (6)
N2—C6	1.315 (5)	C11—H11	0.9500
N2—H2N	1.00 (4)	C12—C13	1.402 (5)
C1—C2	1.352 (5)	C12—H12	0.9500
C1—H1	0.9500	C13—C14	1.376 (5)
C2—C3	1.417 (5)	C13—H13	0.9500
C2—H2	0.9500	C14—C15	1.387 (5)
C3—C4	1.374 (5)	C14—H14	0.9500
C3—H3	0.9500	C15—C16	1.465 (5)
C4—C5	1.455 (5)		
			
C1—S1—C4	91.6 (2)	H7A—C7—H7C	109.5
C9—O2—C10	122.5 (3)	H7B—C7—H7C	109.5
C5—N1—N2	115.1 (3)	C6—C8—C16	120.1 (3)
C5—N1—H1N	123 (3)	C6—C8—C9	120.1 (4)
N2—N1—H1N	113 (3)	C16—C8—C9	119.8 (4)
C6—N2—N1	123.0 (4)	O3—C9—O2	115.1 (3)
C6—N2—H2N	117 (2)	O3—C9—C8	126.4 (4)
N1—N2—H2N	120 (2)	O2—C9—C8	118.5 (4)
C2—C1—S1	112.6 (3)	C11—C10—O2	116.8 (4)
C2—C1—H1	123.7	C11—C10—C15	121.3 (4)
S1—C1—H1	123.7	O2—C10—C15	121.9 (4)
C1—C2—C3	112.5 (4)	C12—C11—C10	118.9 (4)
C1—C2—H2	123.7	C12—C11—H11	120.5
C3—C2—H2	123.7	C10—C11—H11	120.5
C4—C3—C2	112.0 (4)	C11—C12—C13	121.2 (4)
C4—C3—H3	124.0	C11—C12—H12	119.4
C2—C3—H3	124.0	C13—C12—H12	119.4
C3—C4—C5	128.9 (4)	C14—C13—C12	118.4 (4)
C3—C4—S1	111.3 (3)	C14—C13—H13	120.8
C5—C4—S1	119.6 (3)	C12—C13—H13	120.8
O1—C5—N1	121.0 (3)	C13—C14—C15	121.5 (4)
O1—C5—C4	124.1 (3)	C13—C14—H14	119.2
N1—C5—C4	114.9 (3)	C15—C14—H14	119.2
N2—C6—C8	116.7 (4)	C14—C15—C10	118.6 (4)
N2—C6—C7	118.5 (4)	C14—C15—C16	122.5 (4)
C8—C6—C7	124.7 (4)	C10—C15—C16	118.8 (4)
C6—C7—H7A	109.5	O4—C16—C8	123.1 (4)
C6—C7—H7B	109.5	O4—C16—C15	118.6 (4)
H7A—C7—H7B	109.5	C8—C16—C15	118.2 (4)
C6—C7—H7C	109.5		
			
C5—N1—N2—C6	−153.1 (4)	C6—C8—C9—O2	175.9 (3)
C4—S1—C1—C2	−1.1 (3)	C16—C8—C9—O2	−4.3 (5)
S1—C1—C2—C3	1.3 (5)	C9—O2—C10—C11	176.6 (4)
C1—C2—C3—C4	−0.9 (5)	C9—O2—C10—C15	−3.5 (5)
C2—C3—C4—C5	−175.5 (4)	O2—C10—C11—C12	179.0 (3)
C2—C3—C4—S1	0.0 (5)	C15—C10—C11—C12	−1.0 (6)
C1—S1—C4—C3	0.6 (3)	C10—C11—C12—C13	−0.2 (6)
C1—S1—C4—C5	176.6 (3)	C11—C12—C13—C14	0.5 (6)
N2—N1—C5—O1	8.1 (5)	C12—C13—C14—C15	0.4 (6)
N2—N1—C5—C4	−174.8 (3)	C13—C14—C15—C10	−1.5 (6)
C3—C4—C5—O1	148.8 (4)	C13—C14—C15—C16	−178.2 (4)
S1—C4—C5—O1	−26.4 (6)	C11—C10—C15—C14	1.8 (6)
C3—C4—C5—N1	−28.2 (6)	O2—C10—C15—C14	−178.1 (3)
S1—C4—C5—N1	156.6 (3)	C11—C10—C15—C16	178.7 (4)
N1—N2—C6—C8	179.3 (3)	O2—C10—C15—C16	−1.3 (6)
N1—N2—C6—C7	1.2 (6)	C6—C8—C16—O4	−1.3 (6)
N2—C6—C8—C16	−3.4 (5)	C9—C8—C16—O4	178.9 (4)
C7—C6—C8—C16	174.5 (4)	C6—C8—C16—C15	179.6 (3)
N2—C6—C8—C9	176.4 (3)	C9—C8—C16—C15	−0.2 (5)
C7—C6—C8—C9	−5.7 (6)	C14—C15—C16—O4	0.5 (6)
C10—O2—C9—O3	−174.9 (3)	C10—C15—C16—O4	−176.2 (3)
C10—O2—C9—C8	6.2 (5)	C14—C15—C16—C8	179.7 (4)
C6—C8—C9—O3	−2.9 (6)	C10—C15—C16—C8	3.0 (5)
C16—C8—C9—O3	176.9 (4)		

### Hydrogen-bond geometry (Å, °)

**Table:**

*D*—H···*A*	*D*—H	H···*A*	*D*···*A*	*D*—H···*A*
N2—H2N···O4	1.00 (4)	1.64 (5)	2.481 (4)	140 (4)
N1—H1N···O1^i^	0.92 (4)	1.93 (4)	2.841 (4)	177 (4)

Symmetry codes: (i) *x*+1, *y*, *z*.
